# Reaching Out for Inaccessible Food Is a Potential Begging Signal in Cooperating Wild-Type Norway Rats, *Rattus norvegicus*

**DOI:** 10.3389/fpsyg.2021.712333

**Published:** 2021-08-30

**Authors:** Niklas I. Paulsson, Michael Taborsky

**Affiliations:** Division of Behavioural Ecology, Institute of Ecology and Evolution, University of Bern, Bern, Switzerland

**Keywords:** helping, prosocial behaviour, food provisioning, honest signalling, communication, iterated prisoner's dilemma, cooperation, reciprocity

## Abstract

Begging is widespread in juvenile animals. It typically induces helpful behaviours in parents and brood care helpers. However, begging is sometimes also shown by adults towards unrelated social partners. Adult Norway rats (*Rattus norvegicus*) display a sequence of different behaviours in a reciprocal food provisioning task that have been interpreted as such signals of need. The first behaviour in this sequence represents reaching out for a food item the animal cannot obtain independently. This may reflect either an attempt to grasp the food object by itself, or a signal to the social partner communicating the need for help. To distinguish between these two possibilities, we tested in female wild-type Norway rats if the amount of reaching performed by a food-deprived rat changes with the presence/absence of food and a social partner. Focal rats displayed significantly more reaching behaviour, both in terms of number and total duration of events, when food and a potentially helpful partner were present compared to when either was missing. Our findings hence support the hypothesis that rats use reaching behaviour to signal need to social partners that can help them to obtain food.

## Introduction

The ability to comprehend the need of others is widespread in the context of brood care, where variation in offspring begging allows parents to adaptively modulate food provisioning (Grodzinski and Lotem, 2007). Begging signals are frequently used by offspring towards their brood caring parents in mammals (e.g., Kunc et al., 2007; Fröhlich et al., 2020), birds (e.g., Leech and Leonard, 1996) and insects (e.g., Mattey et al., 2018). In contrast it is currently unclear to which extent begging is employed in reciprocal cooperation (cf. de Waal, 2008). If animals respond to the need of prospective receivers of help by increasing their generosity (Schneeberger et al., 2020), this would select for the evolution of signals of demand (Kilner and Johnstone, 1997; Grodzinski and Lotem, 2007), also among unrelated adults (Carter and Wilkinson, 2016; Schweinfurth and Taborsky, 2018a). In fact, great apes have been shown to adjust visual signals depending on how well they seem to understand the intentions of the signaller (Leavens et al., 2005; Cartmill and Byrne, 2007), even if not all studies find support for a response to such signals (Liebal and Rossano, 2017). During reciprocal exchange of goods and services begging can increase the propensity of a previous receiver of help to return the service (Schweinfurth and Taborsky, 2018a). Even without previous helping experience begging signals can provide an incentive to generously donate goods to a social partner in need, which may serve as a first step to establish reciprocal cooperation (Trivers, 1971; Axelrod and Hamilton, 1981; Barta et al., 2011; Roberts, 2020).

Reciprocal altruism or “reciprocity,” where a cost is accepted by an individual to provide a service to a social partner for a delayed benefit, is a mechanism generating evolutionarily stable levels of cooperation between unrelated individuals (Trivers, 1971; Lehmann and Keller, 2006). In the recent past, evidence for enhanced cooperative tendencies of individuals after having received aid from social partners has accumulated in both humans and non-human animals (rats: Rutte and Taborsky, 2007, 2008; Schweinfurth and Taborsky, 2018b,c; bats: Carter and Wilkinson, 2013, 2015; dogs, Gfrerer and Taborsky, 2017, 2018; primates, including humans: Schino, 2007; Jaeggi and Gurven, 2013; Schweinfurth and Call, 2019; birds: St-Pierre et al., 2009; Krama et al., 2012; fish: Croft et al., 2006; Brandl and Bellwood, 2015; for review, see Taborsky et al., 2016, 2021). The propensity to return received favours to social partners may also be modified by the value of previously received service (Dolivo and Taborsky, 2015b; Kettler et al., 2021), the need of prospective receivers (Schneeberger et al., 2012, 2020), and by relatedness among social partners, with kinship affecting reciprocal donations rather negatively (Carter and Wilkinson, 2015; Schweinfurth and Taborsky, 2018c). A question of particular interest in this context is how animals determine the need of prospective receivers, and in turn whether the latter communicate requests to prospective donors (Schweinfurth and Taborsky, 2018a).

A recent study showed that adult Norway rats communicate need to a potentially helpful partner in a reciprocal food-provisioning task (Schweinfurth and Taborsky, 2018a). In 41 out of 50 observed instances involving potential signalling for help in that study, rats in need expressed at least two of three specific behaviours, which appeared in a particular, non-random sequence. These behaviours included reaching out towards the food, emitting ultrasonic calls, and noisy attention grabbing; the behaviours accelerated with increasing need of the recipient (hunger), and they were shown to decrease the latency to food donations provided by the partner. Moreover, prospective receivers displayed the respective next behaviour in the sequence sooner if food donation was delayed, suggesting a sense of urgency communicated to the partner. However, hitherto these alleged signals of need have not been manipulated experimentally in order to test the implied intention of the signaller. This is a serious gap particularly for the first of these three behaviours, “reaching,” which is not directed towards the recipient but to the desired food. It is hence unclear whether it is a signal sent to the potential donor, or merely an inadvertent cue used by the latter. A “signal” implies a behaviour that has been selected for the purpose of communication, i.e., to transmit information, whereas a “cue” corresponds to any feature or trait that can be used by others as a guide to future action (Maynard Smith and Harper, 2003).

Here we aimed to clarify whether the reaching behaviour of Norway rats corresponds to a signal or a cue. We studied female wild-type Norway rats in a reciprocal food-exchange task that was modified from a design used by Rutte and Taborsky (2008). Norway rats are highly social animals (Barnett, 1963; Schweinfurth, 2020) that apply the decision rules of both generalised and direct reciprocity (Rutte and Taborsky, 2007, 2008; Schneeberger et al., 2012; Dolivo and Taborsky, 2015a; Wood et al., 2016; Schweinfurth and Taborsky, 2017, 2018a,b; Delmas et al., 2019; Kettler et al., 2021). Rats have been shown to communicate using vocal (Brudzynski, 2013 for review) and olfactory signals (Gheusi et al., 1997; Moyaho et al., 2015). In the context of reciprocal cooperation, recent studies revealed that rats transfer olfactory information about both their helping behaviour (Gerber et al., 2020) and their current need for help (Schneeberger et al., 2020). Rats were also shown to use visual cues to evaluate challenging tasks (Schneeberger et al., 2012), but the use of visual communication among social partners is currently unclear (Prusky et al., 2000, Dolivo and Taborsky, 2015b).

To distinguish whether reaching out for a food item that cannot be obtained without help from a conspecific is a signal to this social partner, or merely a cue that the latter can use, we experimentally manipulated both the presence of food and the presence of a partner. We measured the number, timing and duration of reaching behaviours of food deprived Norway rats in a setup where either a desired food item that could not be obtained alone, a social partner (potential helper), or both were present in a familiar reciprocal food exchange task (Rutte and Taborsky, 2008), in which one rat can provide food for another, but not for itself. We predicted that if the main purpose of reaching behaviour is to signal a desire for help to a partner, it would be displayed more often or sooner when both food and a partner are present compared to when one of those factors were missing. If the purpose of the behaviour is primarily to acquire the food without assistance, which could also be used as a cue by a partner, we would expect the reaching behaviour to be correlated with the presence of food, regardless of the presence of a potential helper. Finally, if reaching behaviour were a general appeal for support, and not for a particular item, it should be more common in the presence of a partner regardless of the presence of a desirable food item.

## Materials and Methods

### Subjects

Forty-four female Norway rats (source: Animal Physiology Department, University of Groningen, Netherlands) were kept in nine sister groups of five rats each (one of four). Home cages (80 × 50 × 40 cm) contained a wooden house, platform and stick as well as a plastic tunnel, an empty toilet roll, hay, and wood shavings for nesting material. In addition to *ad libitum* access to water and food in the form of conventional rat pellets (except when temporary fasting was required for the experiment, see below), the rats in each cage received fresh food (fruits and vegetables) twice a week and a seed mix four times a week. As rats are nocturnal we employed an inversed 12:12 h light:dark cycle with lights off at 08:00 h to allow us to work during their active period. Artificial red lights were used to enable the observation of the rats during dark hours as they possess a low sensitivity towards red light (Jacobs et al., 2001).

### Pre-experimental Training

All rats were taught to pull a stick for receiving a food reward via a moveable tray following an established protocol (Dolivo and Taborsky, 2015b). As the stick was pulled, a tray containing an oat moved into the cage of the pulling rat. After eight training sessions each lasting 7 mins, 43 rats had learned to perform this task successfully. We used eight successful pulls in one training session as the learning criterion.

In the next training phase, each rat was assigned a partner from their home cage for dyadic training. In this training period no rat ever acquired food for itself by pulling the stick, but it was instead providing food to its partner placed in a neighbouring cage compartment. Over the course of 7 mins the rats took turns first pulling once before the stick was switched to the partner that could then reciprocate the donation, after which the stick was moved back to the first rat. Gradually the number of pulls required before a rat experienced reciprocation was increased. Subsequently a time delay was introduced between reciprocation periods, which was stepwise increased to 24 h. In between training sessions the test rats were returned to their home cages. After 18 training sessions 40 rats had learned to perform this task successfully.

### The Moveable Tray

**Figure 1:**
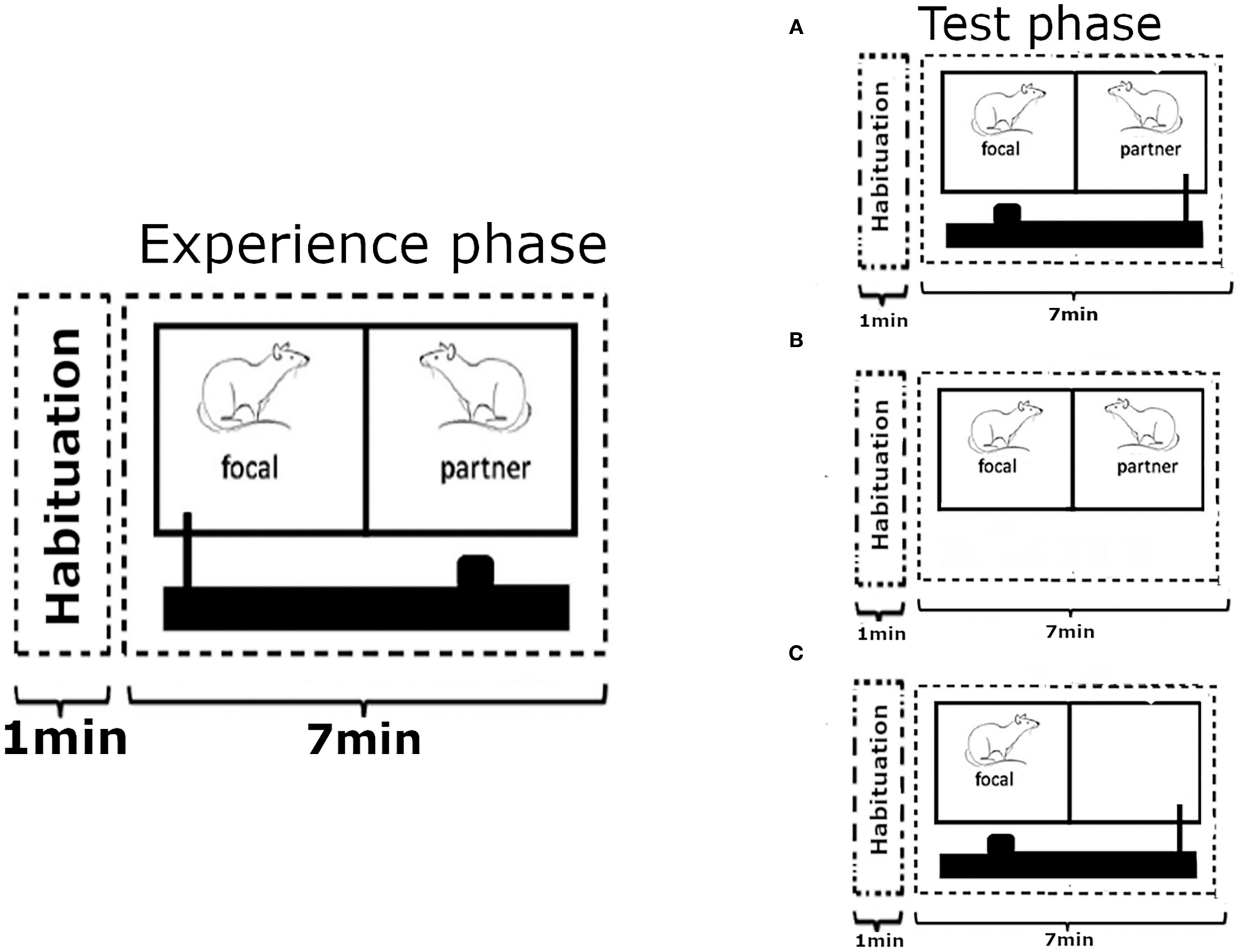
Outline of the experimental design. Each test phase was preceded by an experience phase where the focal animal could provide food to its partner an undetermined number of times, over 7 mins, by pulling a baited tray with a wooden stick towards the cage. On the following day, after 18 h of food deprivation, the focal animal was returned into the experimental cage where the roles were reversed and **(A)** the partner rat could now provide food to the focal subject. In the first control condition **(B)** the partner from the previous day was present in the neighbouring cage compartment, but no moveable tray with food was present. In the second control condition **(C)** the focal rat was on the receiving side of the food tray like in condition **(A)**, but no partner was present to move it. Each focal animal went through all three experimental treatments in a random order, each time preceded by the experience phase with a new partner on the previous day. Figure modelled on comparable depiction in Schweinfurth and Taborsky (2018a).

The tray consisted of a PVC sheet mounted to rails with ball bearings allowing it to be moved with minimal resistance. On opposite ends of the front of the tray, two wells were placed to hold food items, which prevented the food from sliding when the tray was pulled. On the outer side of each well, a small plastic tube was attached to act as an anchor point for a stick that could be pulled to move the tray (see sketch in Figure 1). A Raspberry Pi 3B computer in combination with a small limit switch attached to the base of the platform was used to record the exact time at which the tray had been pulled to the maximum extended position where the food could be reached by the receiver. Following a 10 s delay, a servo arm controlled via a remotely powered 16-channel, 12-bit PWM Fm+ I2C-bus LED controller (PCA9685) moved the tray back and held it in a locked position for 2 s, to allow a new food item to be placed on the tray by the experimenter. Then the tray could be moved again by the experimental subjects. At the end of each trial, the servo arm moved the tray back to the locked position to mark the end of the observation and prevent further pulling.

### Test Procedure

The experimental design followed the procedure of Schweinfurth and Taborsky (2018a), where rats were enabled to reciprocate food donations to a previously helpful partner that was now food-deprived. Each experimental treatment started with an experience phase during which a focal animal (*N* = 25) was paired up with an unrelated and unfamiliar individual, to avoid confounding effects of relatedness and previous social interactions. The focal rat could then provide the partner with food by pulling a stick connected to the moveable tray, over the course of 7 mins (Figure 1). Thereafter, both rats were returned to their respective home cages and the food was removed from the cage of the focal rat to increase the likelihood of reaching behaviour in the subsequent test phase (Schweinfurth and Taborsky, 2018a). The order at which rats from different cages were tested was randomised, as was the order of focal animals from within each cage. At no time was a partner rat food-deprived as part of the test procedure, and partner rats that shared their home cage with a focal rat were always given a minimum of 36 h of free access to food prior to being used.

Eighteen hours after the removal of the food from the home cage of the focal animal it was returned to the test cage to undergo one of three treatments for 7 mins. In the “food present” treatment the focal animal was put on the receiving side of the moveable tray, unlike in the experience phase where it had played the part of the provider, and no partner was present to move the tray to fetch the food for the focal rat. In the “partner present” treatment, the partner from the previous experience phase was present in the adjacent cage compartment, but no moveable tray with food was presented. In the “food and partner present” treatment, both a moveable tray with food and the partner from the previous experience phase to operate it were present (Figure 1). The same partner rat was never used for more than one treatment to retain the unfamiliarity status, and the position of the focal rat within the test cage was randomised to avoid potential side bias. Each focal rat was tested once for each treatment in a random order. The experiment extended over 3 weeks, and each focal animal was used for testing only once per week. Experience phases took place always on a Tuesday or Thursday, leaving 4–6 days between a test phase and the experience phase of the next treatment for each focal rat.

### Behavioural Data

Each trial was video-recorded using a handycam with night vision-mode (Sony HDR-CX550). From these recordings the numbers, beginnings and ends of all reaching behaviours were scored at a 0.2 s resolution. The total number of food items donated in both experience and test phases were recorded automatically by the Raspberry Pi 3B computer. The rats would pull the stick either with their teeth (more often), or with their forelimbs (rarer), and we considered a rat to be reaching when either the mouth or forelimbs were being held outside the cage through the gap in the cage bars where the food tray would enter (estimated maximum distance reached: 1 cm for mouth, 4 cm for forelimbs). These behaviours were easily identifiable with recordings taking place from a mostly top-down view, allowing the bars of the cage to act as a line that, if crossed by forelimbs or nose, was interpreted as reaching. Any pause in reaching longer than 0.5 s was considered to mark the end of that reaching bout. All video recordings were analysed by the same person (NP). Ten videos were re-analysed to assess intra-observer consistency, and found no difference in the number of reaching behaviours observed, and agreement in the duration of 92% (44/48) observed reaching behaviours. Additionally, a bat detector (Pettersson 1000X) was used to record all ultrasonic vocalisations by the focal rats during testing to be used in a concomitant study.

### Statistical Analyses

All statistical analyses were performed using R (Version 4.0.2 R Core Team, 2020; packages “lme4,” “lmtest,” “MASS,” “survival,” “outliers”), applying a significance criterion of *p* < 0.05 as standard.

To test whether reaching behaviour differed between treatments with or without food and/or a partner, we analysed (i) number of reaching events using general linear mixed models (GLMM) assuming a negative binomial distribution, and (ii) total duration of reaching using a GLMM assuming a gamma distribution with a log-link function. Our initial models included the following fixed effects: treatment (levels: Food present, Food and Partner present, Partner present; using Food present as the control treatment), number of stick pullings performed by the focal rat in the experience phase (range: 0–15), and number of stick pullings performed by the partner rat in the test phase (confined to the “food and partner present” treatment; range: 0–17). As each focal rat was used multiple times, the ID of the focal animals grouped by housing cage was included as random factor. Partner ID was included as a random factor in the analysis of reaching duration, but not of the number of reaching events due to low variance (variance: 1. 8 × 10^−14^, SE: 1.039 × 10^−7^). The full models were tested against null models using only intercept and random effects with a log-likelihood test to validate that key factors improved model fit. Using the drop1 function from the “lme4” package the number of pulls in the test phase was dropped from both models as this improved the AIC by at least two. A Grubbs-test from the “outliers” package was used to test for statistical outliers.

To test for treatment effects on the latency until the first reaching behaviour was shown by the focal rats we utilised a Cox proportional hazard model (CPHM; function “coxph”). We estimated the model-predicted survival probability using focal animal ID as frailty random effect assuming a gamma distribution following, (Landes et al., 2020).

As rats with access to the pulling stick would occasionally move the tray only part of the way required for the food item to be reached by their partner, the latter were sometimes able to complete the movement of the tray by reaching out and grabbing it. In 6 out of 25 test trials of the “food and partner present” treatment this occurred before the first reaching behaviour had been shown. These six observations were not considered for the analysis of latencies to first reaching behaviour (*N* = 19), because the response of the receiver could not be unequivocally interpreted.

## Results

### Number of Reaching Behaviours

**Figure 2:**
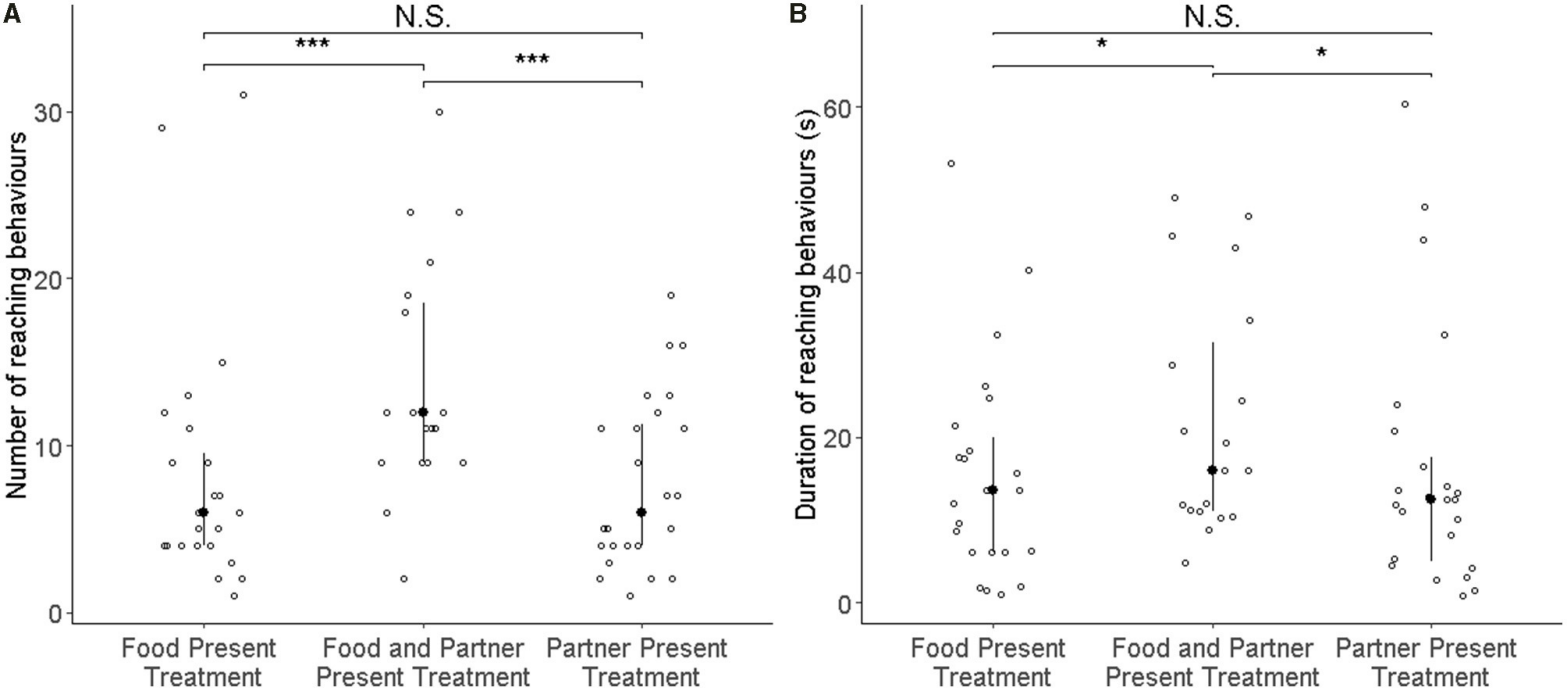
Number of reaching events **(A)** and the total duration of all reaching behaviours **(B)** of all focal rats (*N* = 25) in the test phase. Open circles represent individual data points, filled circles the median value, and whiskers the interquartile ranges. In the three treatments, either food or the partner were absent (left and right columns), or both were present (middle). Both the number of reaching events and their total duration increased significantly in the presence of both food and partner relative to the two control conditions as denoted by asterisks (***p < 0.001, *p < 0.05).

**Figure 3:**
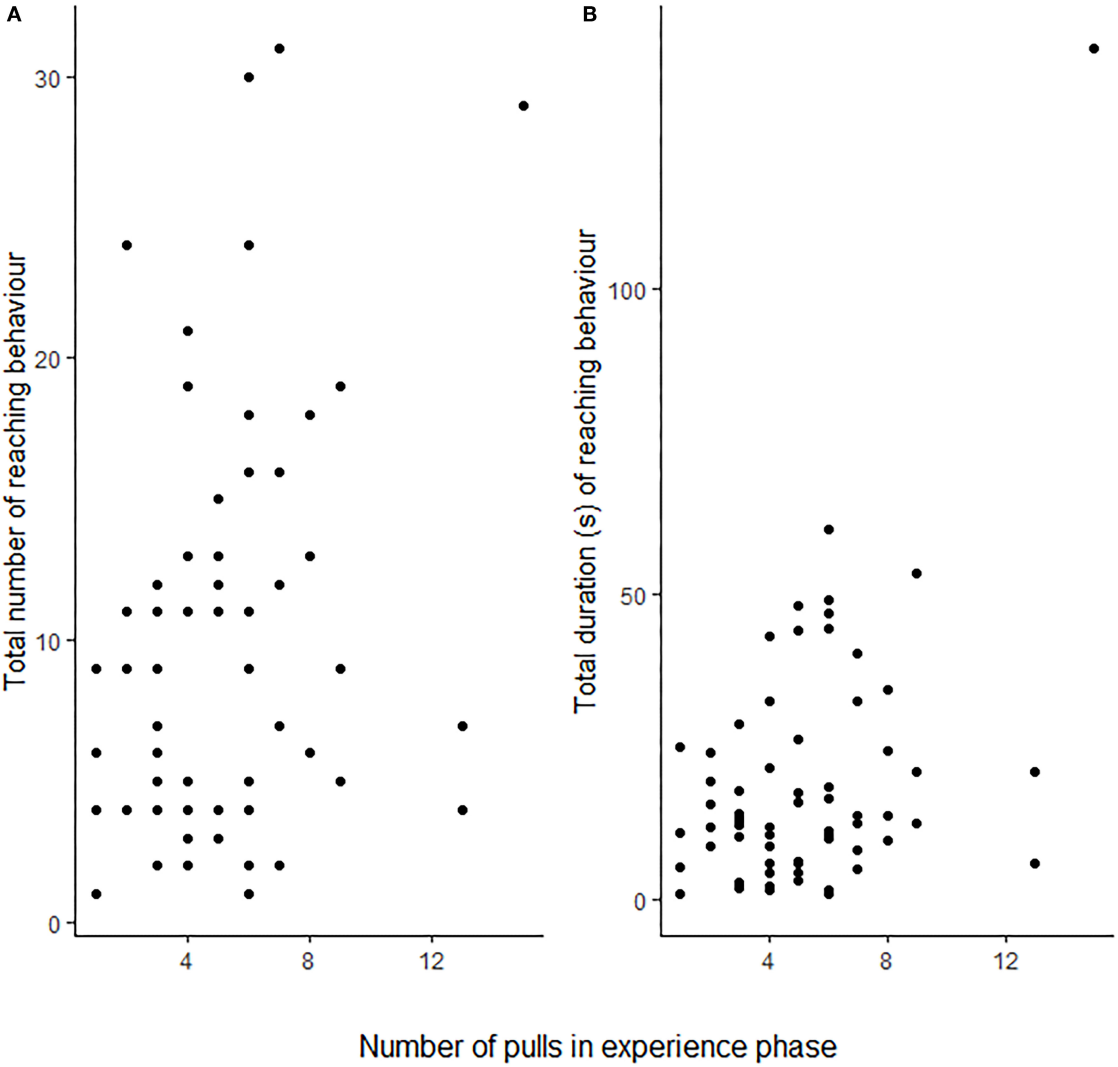
The focal rats' (*N* = 25) total number of reaching events **(A)** and total amount of time spent reaching **(B)** in the test phase in relation to the number of food provisionings the same focal animal performed during the test phase. Each point corresponds to a single individual. For statistical results see Table 1.

Rats (*N* = 25) showed more reaching behaviours when a partner capable of providing food was present than when none was present (i.e., Food treatment; GLMM: ß = 0.887 ± 0.164 SE, *p* ≤ 0.001), but not in the presence of only a partner without food that it could have fetched for the focal subject (GLMM: ß = 0.083 ± 0.170 SE, *p* = 0.624; Figure 2A; Table 1). Additionally, the number of reaching events was significantly higher in focal rats that had pulled more often for their partner in the experience phase (ß = 0.064 ± 0.026 SE, *p* = 0.016; Table 1; Figure 3).

**Table 1:** Effects of experimental treatment and previous helpful acts on (A) the number and (B) duration of reaching events, using GLMMs with a negative binomial or gamma distribution (log-link), respectively.

**(A)**	**Number of reaching events**	**Estimate**	**SE**	**Z**	***p***
	Intercept	1.587	0.207	7.666	** <0.001**
	Food and partner treatment	0.887	0.164	5.391	** <0.001**
	Partner treatment	0.083	0.170	0.490	0.624
	Pulls in experience phase	0.064	0.026	2.419	**0.016**
**(B)**	**Total duration of reaching**	**Estimate**	**SE**	***t***	***p***
	Intercept	2.206	0.282	7.832	** <0.001**
	Food and partner treatment	0.729	0.207	3.513	** <0.001**
	Partner treatment	−0.068	0.210	−0.324	0.746
	Pulls in experience phase	0.070	0.038	1.867	0.062

*Both models used Focal ID [model (A) N = 25; model (B) N = 24] as a random factor, with (B) also using Partner ID as a second random factor. All treatments are in comparison to the “food present” treatment. Significant p-values are marked in bold, non-significant trends are underlined*.

### Total Duration of Reaching

One observation from the food-only treatment was considered an outlier by the Grubbs test (*p* < 0.001) and was not considered for the observation (*N* = 24). As with the number of reaching behaviours, the total duration of reaching by focal rats in the presence of a partner and food was significantly longer than when no partner was present (i.e., Food treatment; GLMM: ß = 0.729 ± 0.207 SE, *p* < 0.001; Figure 2B; Table 1B), but this was not the case when only a partner was present without food it could have fetched for the focal subject (GLMM: ß = −0.068 ± 0.210 SE, *p* = 0.746). The number of food donations performed by the focal animal in the experience phase showed a non-significant trend to correlate positively with the total amount of reaching in the test (GLMM: ß = 0.070 ± 0.038, *p* = 0.062).

### Latency to First Reaching Behaviour

Kapplan-Meier conditional probabilities estimated the mean time of all first reaching behaviours (*N* = 19) at 27 s from start of the experiment. The distribution of censored and uncensored data was deemed acceptable for continued analysis, which revealed that the latency to the first reaching behaviour changed significantly with the presence of both food and a partner (CPHM: ß = −0.898 ± 0.356 SE, *p* = 0.012; HR = 0.2.454; 95% CI of HR = 1.222–4.925), but not with partner alone (CPHM: ß = −0.114 ± 0.297 SE, *p* = 0.701; HR = 0.892; 95% CI of HR = 0.499–1.597) when compared to the control treatment with only food present.

## Discussion

In this study we investigated whether reaching out for inaccessible food corresponds to a signal of need for help in wild-type Norway rats. In accordance with the hypothesis that reaching is an intentional signal serving to elicit help by a social partner, we found a significant increase in both the number of reaching behaviours and the total duration for which this behaviour was shown, as well as a decrease in the latency to its first occurrence, when both food and a partner were present compared with a situation where either was missing. Our data do not support the two alternative hypotheses we tested, namely that reaching corresponds to a general signal for help, or that it reflects merely a self-serving attempt to reach the inaccessible desideratum. This is all the more remarkable because in this experimental test, which followed a phase in which the rats had supplied a partner with food, they experienced a situation for the first time in which either a partner to pull food for them, or food to be fetched, were missing. Regardless, the latency to start reaching was shorter when both food and partner were present compared to when there was no partner available to provide food, further substantiating that rats alter their reaching behaviours depending on whether or not a partner is present to provide help.

In addition to the clear effect of the presence of food and a partner, reaching was also shown significantly more often by rats that had performed a higher number of food donations to their partner in the preceding experience phase. This suggests some contingency regarding the propensity of a rat to help a partner and its expectancy of a restitution. Norway rats have indeed been shown to return more help to previously more helpful individuals in a similar food provisioning task (Kettler et al., 2021), and to modify their help also in response to the quality of help they received (Dolivo and Taborsky, 2015b). Rats were shown to switch between alternative roles also in other turn-taking games (Reinhold et al., 2019), and neurological evidence suggests that rats possess rudimentary capabilities to predict forthcoming events (Seamans et al., 2008). It seems possible, therefore, that rats providing more help to a partner in a turn-taking game have a higher expectation of a socially mediated return benefit in the subsequent phase, similar to anticipation effects as known from conditioned tasks (Bolles and Moot, 1973).

Norway rats are nocturnal animals that obviously rely less on visual stimuli than diurnal species, and previous studies have shown that rats make use particularly of auditory (Blanchard et al., 1991; Brudzynski and Ociepa, 1992; Brudzynski, 2005; Pereira et al., 2012) and olfactory (Brown, 1979; Gheusi et al., 1997; Moyaho et al., 2015; Schneeberger et al., 2020) information from conspecifics. In an experimental setup similar to ours, visual cues have indeed turned out to be of little importance for successfully performing reciprocal food exchanges (Dolivo and Taborsky, 2015b). So it seems puzzling that reaching out towards something, which appears to be primarily a visual signal, elicits a helpful response in a receiver of such signal, as has been demonstrated in a previous study (Schweinfurth and Taborsky, 2018a). In general, signals are considered to be mechanically ineffective behaviours, unable to accomplish the desired goal (e.g., Pika and Bugnyar, 2011), but this does not mean that mechanically effective behaviours cannot be used as signals in a different context. In our case, a food fetching behaviour is shown by Norway rats in a situation where only a social partner can provide food to them, i.e., where the behaviour is mechanically ineffective, and apparently they use this behaviour mainly when both food and partner are available. Whether the visual component of this behaviour is indeed recognised by the signal receivers, or its inevitable or intended correlates in another sensory modality, poses an interesting question for future studies. In the context of food provisioning to social partners, Norway rats have been shown to respond to the odour produced by a cooperating conspecific (Gerber et al., 2020), and they adjust their helpfulness to the hunger state of social partners merely based on olfactory information (Schneeberger et al., 2020). Therefore, it seems possible that the reaching behaviour shown in this study may also coincide with the emission of odour that can be more easily detected by signal receivers than the visual feature. In fact, a combination of cues of need by a partner could be used to pinpoint who is signalling for help in a large colony where movement of air and individuals may make it difficult to locate the exact origin of a particular scent. The production of acoustical signals concurrently with the reaching behaviour might be another possibility, and the reaching behaviour itself may be detectable also by auditory means, which would provide alternative ways of signal transmission.

In conclusion, our study provides evidence that Norway rats enhance reaching behaviour in the presence of a partner and food the latter can deliver to them, as expected if it is used as a signal a need for help to social partners. In connection with a previous study showing that this behaviour indeed triggers help in a receiver of the signal (Schweinfurth and Taborsky, 2018a), reaching out for an inaccessible item seems to be part of the communication system of these highly social animals. Future studies should unveil which sensory modality involved in this signal conveys the most critical information. Furthermore, our data revealed that there is a quantitative contingency between the helpfulness of a rat and the number of reaching behaviours shown, which might suggest an expectation of return benefits. This is in accord with previous results showing the inverse relation: that rats accredit more to social partners that have provided more or better service to them before (Dolivo and Taborsky, 2015b; Kettler et al., 2021).

## Data Availability Statement

The raw data supporting the conclusions of this article are available in the Supplementary Material.

## Ethics Statement

The animal study was reviewed and approved by Veterinary Office of the Canton of Bern (Licence Number BE55/18).

## Author Contributions

NIP and MT designed the study. NIP performed the experiments and analysed the data. NIP and MT both contributed to the design of the data analysis and jointly wrote the manuscript. All authors contributed to the article and approved the submitted version.

## Conflict of Interest

The authors declare that the research was conducted in the absence of any commercial or financial relationships that could be construed as a potential conflict of interest.

## Publisher's Note

All claims expressed in this article are solely those of the authors and do not necessarily represent those of their affiliated organizations, or those of the publisher, the editors and the reviewers. Any product that may be evaluated in this article, or claim that may be made by its manufacturer, is not guaranteed or endorsed by the publisher.
